# The ongoing need for rates: can physiology and omics come together to co-design the measurements needed to understand complex ocean biogeochemistry?

**DOI:** 10.1093/plankt/fbac026

**Published:** 2022-06-08

**Authors:** Robert F Strzepek, Brook L Nunn, Lennart T Bach, John A Berges, Erica B Young, Philip W Boyd

**Affiliations:** Australian Antarctic Program Partnership (AAPP), Institute for Marine and Antarctic Studies, University of Tasmania, 20 Castray Esplanade, Hobart, TAS 7004, Australia; Department of Genome Sciences, University of Washington, Foege Building S113 3720 15th Ave NE, Seattle, WA 98195, USA; Institute for Marine and Antarctic Studies, University of Tasmania, Hobart, TAS 7004, Australia; Department of Biological Sciences and School of Freshwater Sciences, University of Wisconsin-Milwaukee, 3209 N. Maryland Avenue, Milwaukee, WI 53211, USA; Department of Biological Sciences and School of Freshwater Sciences, University of Wisconsin-Milwaukee, 3209 N. Maryland Avenue, Milwaukee, WI 53211, USA; Australian Antarctic Program Partnership (AAPP), Institute for Marine and Antarctic Studies, University of Tasmania, 20 Castray Esplanade, Hobart, TAS 7004, Australia; Institute for Marine and Antarctic Studies, University of Tasmania, Hobart, TAS 7004, Australia

**Keywords:** physiology, omics, co-design, marine biogeochemistry, ocean change

## Abstract

The necessity to understand the influence of global ocean change on biota has exposed wide-ranging gaps in our knowledge of the fundamental principles that underpin marine life. Concurrently, physiological research has stagnated, in part driven by the advent and rapid evolution of molecular biological techniques, such that they now influence all lines of enquiry in biological oceanography. This dominance has led to an implicit assumption that physiology is outmoded, and advocacy that ecological and biogeochemical models can be directly informed by omics. However, the main modeling currencies are biological rates and biogeochemical fluxes. Here, we ask: how do we translate the wealth of information on physiological potential from omics-based studies to quantifiable physiological rates and, ultimately, to biogeochemical fluxes? Based on the trajectory of the state-of-the-art in biomedical sciences, along with case-studies from ocean sciences, we conclude that it is unlikely that omics can provide such rates in the coming decade. Thus, while physiological rates will continue to be central to providing projections of global change biology, we must revisit the metrics we rely upon. We advocate for the co-design of a new generation of rate measurements that better link the benefits of omics and physiology.

## INTRODUCTION

A major challenge for ocean scientists is to address key questions on future ecosystem services. For example, how will global climate change alter low latitude primary productivity and hence food security? A powerful tool to address these global-scale questions is Earth system models, such as those within the Coupled Model Intercomparison Project (CMIP6) (Kwiatkowski *et al*., 2020). The CMIP currencies include the critically important rates at which metabolism occurs in living organisms (i.e. physiological rates) and the biogeochemical fluxes of bioactive elements. It is unlikely that these currencies will change in the coming decade, for example when CMIP7 is developed. Currently, the accuracy of the model projections is hindered by two issues: (i) computational limitations to developing more complex parameterisations for processes such as nitrogen (N) fixation (Kwiatkowski *et al*., 2020) and (ii) our inability to untangle how marine life responds to complex ocean change (Weisse and Montagnes, 2021). For the latter, we need to decipher the fundamental physiological rules that govern biological responses to ocean change. These include the metabolic co-dependencies in response to multiple stressors, and strategies to buffer responses to rapid change, such as phenotypic plasticity and microevolution.

The physiological metrics used to quantify biological rates that are the cornerstones of Earth system models, such as primary productivity, have not fundamentally changed in decades. In contrast, omics techniques have evolved rapidly this century and have the potential to supersede physiological metrics as the main approach to study the fundamental principles driving marine life. With this dominance has come an implicit assumption by some that measuring physiological rates directly is obsolete, as they can be inferred from omics (Hellweger, 2020; McCain *et al*., 2021). However, omics provides a surfeit of data, at a level of detail that is often difficult to relate to the information provided by physiological rate measurements and the current needs of Earth system models. This growing mismatch between the currencies of global-scale models (rates and fluxes) and the aspirations of omics (coupling cellular potential via omics to Earth system model projections) must be addressed urgently.

**Fig. 1:**
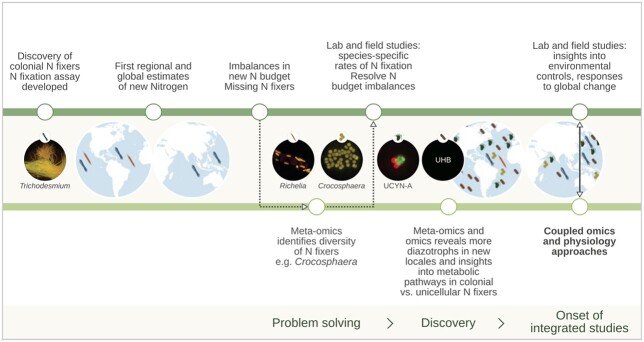
The contributions of physiology and omics to understanding the role of diazotrophy in the ocean N cycle (based on Zehr and Capone, 2020). Key events in the physiology timeline (top green line) include the estimation of N fluxes through nitrogenase activity (Dugdale and Dugdale, 1962), initial estimates of global marine N_2_ fixation rates (Capone *et al*., 2005) and the combining of lab and field measurements to understand individual diazotrophs and community contributions and constraints. Pivotal events in the omics timeline (lower green line) include problem solving (Zehr and Montoya, 2007) and discovery of diazotroph diversity including in unicellular cyanobacteria group A (UNCYN-A) and diverse uncultured heterotrophic bacteria (Martínez-Pérez *et al*., 2016). Recent examples of more integrated physiological and omics co-designed studies (Walworth *et al*., 2016; Qu *et al*., 2022) offer an important way forward.

Here, we ask: How do we translate the wealth of information on physiological *potential* from omics-based studies to quantifiable physiological rates and, ultimately, to biogeochemical processes and their representation in Earth system models? We employ three approaches to address this question. First, we use ocean N_2_ fixation as an illustrative example of research that has evolved through joint advances in physiology and omics (Fig. 1). Second, we examine the recent trajectory of biomedical research to forecast how ocean sciences might evolve in the next decade. Third, we broaden our view by examining insights that can be gained for understanding the ocean phosphorus (P) and iron (Fe) cycles by better linking omics and physiology. We conclude with advocacy for the co-design of better physiological tools.

## LESSONS FROM MARINE DIAZOTROPHY

Here, we use the history of N_2_ fixation (diazotrophy) research to reveal the benefits and limitations of physiological rate measurements, and how these measurements are complemented by more recent omics approaches (Fig. 1).

The contribution of diazotrophy to the supply of new N is an important facet of the ocean N cycle (Fogg, 1942; Dugdale *et al*., 1961). Physiological studies played an important early role by quantifying rates of diazotrophy (e.g. Dilworth, 1966). These measurements provided the integrated rates necessary to estimate global biogeochemical fluxes of N (Karl *et al*., 2002) and to identify the environmental drivers of N_2_ fixation (see Carpenter and Capone, 2008), including how climate change may alter future diazotrophy (Garcia *et al*., 2015; Hutchins *et al*., 2013) leading to improved model projections (see Fig. 3 in Hutchins *et al*., 2013). Still, imbalances in these N fluxes have uncovered unidentified N sources, and the subsequent application of genetic tools has identified additional diazotrophic taxa that contribute to ocean N_2_ fixation (Zehr and Capone, 2020).

Nitrogen fixation provides clear examples of both the limitations and benefits of non-targeted omics-based discoveries (Fig. 1). Nitrogenase (*nif*) genes can be used to detect N_2_ fixation potential, and their expression is used as an index of N_2_ fixation activity (Zehr *et al*., 1996; Zehr and Montoya, 2007). Omics has revealed diverse N_2_ fixers including the unicellular cyanobacteria *Crocosphaera* and UCYN-A, and endosymbiotic and heterotrophic diazotrophs (Mehta *et al*., 2003; Church *et al*., 2005; Martínez-Pérez *et al*., 2016). But, *nif* gene abundance does not directly equate to either diazotroph abundance- and biomass-based biogeographic “currencies” (Meiler *et al*., 2022) or to N_2_ fixation rates (Turk-Kubo *et al*., 2013). Transcriptomics and proteomics targeting *nif* genes provide more relevant information about nitrogenase *activity* than genomics. However, taxon-specific dynamics can complicate the estimates of community N_2_ fixation rates (Church *et al*., 2005), and measurements of *nif* expression are not well correlated with ^15^N-based rates of N_2_ fixation (Turk *et al*., 2011).

Thus, despite the insights gained from omics, critical gaps remain in our understanding of the phylogenies, distribution and physiology of marine N_2_ fixers, and accurate global estimates of N_2_ fixation remain elusive (Zehr and Capone, 2020). Measuring N_2_ fixation remains critical to estimate the biogeochemical processing and ecological fates of new N. Yet, N_2_ fixation is not included in the CMIP6 models, which currently project declining productivity in low latitude oceans in coming decades (Kwiatkowski *et al*., 2020). Therefore, both rates and omics will be needed increasingly to reveal and quantify currently unknown (but biogeochemically important) pathways for the turnover of N (Fig. 1) to improve global models.

Resolving these unknowns will require combined measurements of *nif* gene expression with rate measurements based on nitrogenase enzyme activity (e.g. Turk *et al*., 2011). Broader application of flow-through high-throughput rate measurements can improve the spatial and temporal coverage of N_2_ fixation (Cassar *et al*., 2018). Rates, when coupled with omics approaches to N_2_ fixation research (Tang *et al*., 2020), will continue to expand our understanding of diazotroph diversity and could help focus N_2_ fixation rate measurements on these emerging diazotrophic groups (Zehr and Capone, 2020). Mechanistic controls on diazotrophy can be revealed through variations in *nif* gene expression (Church *et al*., 2005), supporting prior conclusions that local environmental conditions influence N_2_ fixation rates (Capone, 1993; Carpenter and Capone, 2008). Such environmental controls could be further explored using targeted proteomics analyses (e.g. Saito *et al*., 2011).

The history of N_2_ fixation research clearly demonstrates the rapid progress that can be made by bringing together omics and physiology. Walworth *et al*. (2016) in a lab-based investigation of long-term (i.e. multi-year) adaptation by *Trichodesmium* to high carbon dioxide levels demonstrated that genetic assimilation may be driving adaptation, but also identified the metabolic pathways that were initially altered and then maintained during this adaptation. In another lab-based diazotroph study, Qu *et al*. (2022) provide a mechanistic understanding of how the diazotrophs *Crocosphaera* and *Trichodesmium* acclimate or adapt to ocean global change using phenotypic metrics in conjunction with whole-genome sequencing and variant analysis.

**Fig. 2:**
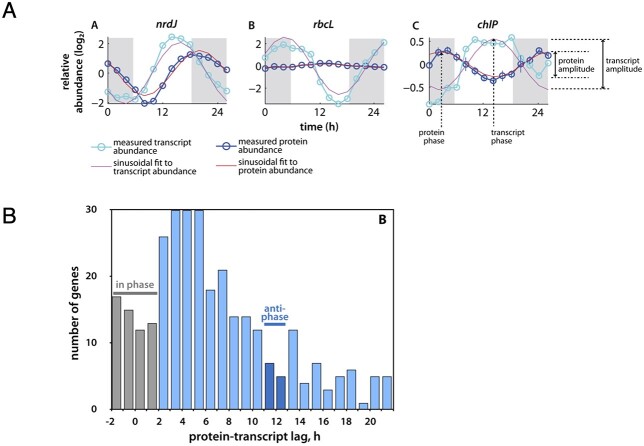
Examples of the potential for mismatches in transcriptomics versus proteomics in a pico-prokaryote over the diurnal light:dark cycle. (**A**) The diel cycling and amplitudes of transcripts and proteins in *Prochlorococcus* for Ribonucleotide reductase (nrdJ), the large sub-unit of Rubisco (rbcL) and Geranylgeranyl diphosphate reductase (chlP). (**B**) Histogram of lag-times for proteins and their transcripts for a 312 gene dataset. Antiphase refers to genes that are offset by ~12 h (i.e. 50%) of the diel cell cycle. Redrawn from Waldbauer *et al*. (2012).

## THE STATUS OF OMICS

Both marine and biomedical sciences study the genome, transcriptome, proteome and metabolome, with most research on the first three. In the field of meta-omics (where “meta-” refers to a population of organisms rather than a single organism), marine metagenomics has set the pace and is directly influencing research into the human microbiome (Poceviciute and Ismagilov, 2019). Here, we focus on genomics through to proteomics at the cellular level where, in contrast to meta-omics, biomedical research has led the way (Okada and Kuroda, 2019). Genomics is the most distal to physiological activity. It demonstrates the breadth of possible gene functions but only catalogues the functional potential of an organism (Sunagawa *et al*., 2015). Transcriptomics is a popular approach to explore how organisms respond to environmental change by characterizing shifts in mRNA abundance (Evans, 2015). Feder and Walser (2005) offered a pointed description of the major issues facing the use of transcriptomics in finding the genes that matter for environmental adaptation. Their critique concentrated on three major issues: (i) genes with large impacts on fitness are rare and therefore unlikely to be identified with transcriptomics, (ii) the relationship between gene expression and fitness is unreliable and (iii) fitness is primarily determined by proteins, and mRNA abundance is a poor proxy for protein abundance. Proteomics, on the other hand, provides taxonomically specific information on metabolic enzymes and is therefore more proximal to physiological activity. Proteomics has advanced methodologically, with more accurate standardized quantitative analyses (Collins *et al*., 2017; Pino *et al*., 2020) and protein identifications that allow metabolic profiling (Nunn *et al*., 2013; Mikan *et al*., 2020).

**Fig. 3:**
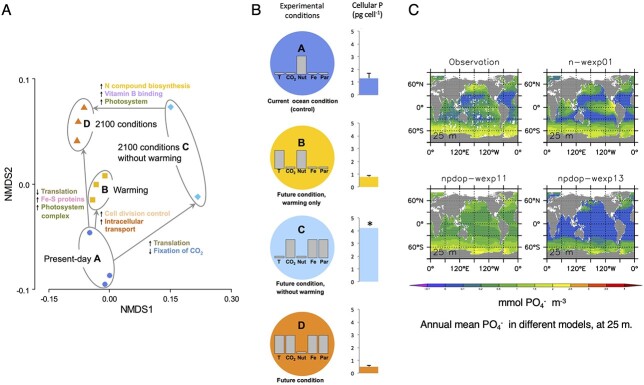
An example illustrating the utility of physiological metrics as a “currency converter” to link omics and biogeochemical modeling. (**A**) Higher (downward arrows) and lower expression (upwards arrows) of proteins in 4 treatments within a climate change manipulation experiment measured with proteomics (Boyd *et al*., 2015). Warming results in higher expression of P-containing proteins associated with translation. (**B**) Corresponding changes to the cellular P quotas (no change in cell size was observed) of the study subject, a lab culture of a subantarctic diatom, across the treatments A–D. This physiological metric reveals the causal link between lower expression of translation proteins and decreased P quotas (as previously described by Toseland *et al*., 2013). (**C**) A subset of global model projections of upper ocean phosphate (PO_4_^−^) stocks across biogeochemical models of different complexity (Kriest *et al*., 2010). The approaches employed in panels A and C can be linked using the cellular P quotas obtained from panel B.

Numerous efforts have been made to identify correlations between omics layers. However, evidence from both marine and biomedical science reveals that making these linkages is not straightforward. For example, in marine sciences, it is well recognized that the amplitude and timing of the mRNA pool does not align with protein expression. This misalignment was illustrated in Waldbauer *et al*. (2012) while tracking diel changes in the transcriptome and proteome within a single cyanobacteria species (Fig. 2). Subsequent research on the diatom *Phaeodactylum tricornutum* used multiple omics layers to explore the regulation of N limitation and again reported mismatches between transcript, protein and metabolite abundance (Remmers *et al*., 2018). In the further advanced biomedical field, it remains difficult to obtain mechanistic and functional insights by simply integrating multiomics data (Okada and Kuroda, 2019). As far back as the late eighties, Kurland and Ehrenberg (1987) discussed the challenges of linking cellular design and molecular design (such as via enzyme expression) in the context of physiology. More recently, Lalanne *et al*. (2018) uncovered post-transcriptional controls that ensure the maintenance of the protein stoichiometries required for specific biological pathways. This compensatory mechanism rectifies divergences in regulation driven by changes of internal promoters and terminators. Hence, even in advanced biomedical research, there are confounding issues, driven by post-transcriptional and post-translational modifications to enzymes, in deriving metabolic rates from omics.

In the marine context, omics has clearly demonstrated large-scale patterns in microbial diversity across oceanic provinces and provided insights into which metabolic pathways are active (Fig. 1). But, omics-based approaches provide static “snap-shots” of physiological potential, and we need to improve our quantitative, process-level understanding of the roles of marine microbes in biogeochemical cycles. Indeed, it is physiological activity—as modified by biological species differences, environmental drivers and the interactions between the two—that ultimately drives biogeochemical cycles.

**Fig. 4:**
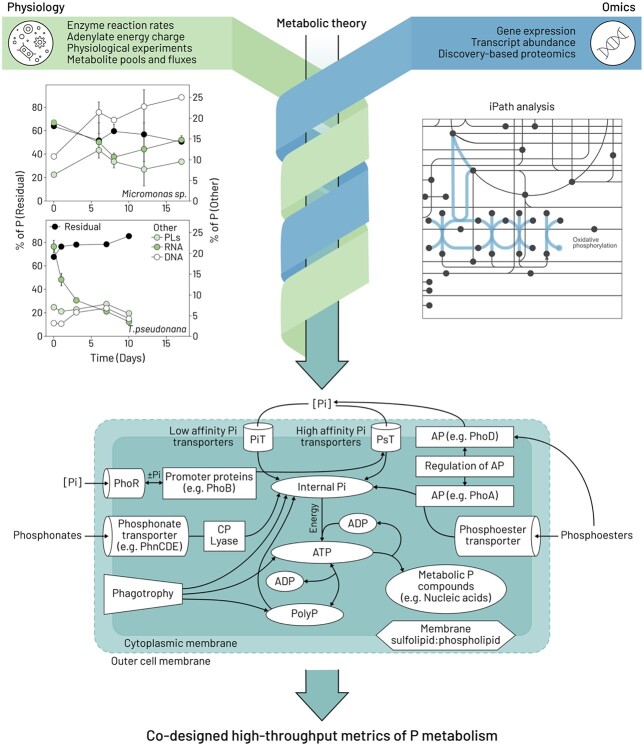
The potential of reverse-engineering physiological metrics to provide better linkages with molecular tools using the example of phosphorus (P). Findings from a physiological study (top left; redrawn from Liefer *et al*., 2019. PLs = phospholipids) using a cluster of long-established metrics (residual P pools/intracellular storage of inorganic P) to compare the P allocation strategies of a diatom (*Thalassiosira pseudonana*) and a prasinophyte (*Micromonas sp*.). Other physiological approaches (top left green box) include measuring enzyme reaction rates, clever experimental design (Bell, 2019), quantifying adenylate energy charge (Karl, 1980) and tracking metabolite pools and fluxes (Moran *et al*., 2022). The top right figure is a Kyoto Encyclopedia of Genes and Genomes (KEGG) map from Interactive Pathways Explorer v3 (iPath) (https://pathways.embl.de; Letunic *et al*., 2008; Darzi *et al*., 2018). iPath is a web-based tool for the visualization and analysis of cellular pathways from omics (e.g. see Nunn *et al*., 2013 for Fe replete versus Fe deplete proteomes). In this example, the search terms “phosphate” and “oxidative phosphorylation” returned compound C00009 (https://www.genome.jp/dbget-bin/www_bget?C00009) and map00190 (https://www.genome.jp/dbget-bin/www_bget?map00190), respectively. The results are shown in the iPath panel as the blue dot (phosphate; C00009) and the blue line (map00190) connecting compounds (nodes) with enzymes (lines). Combining approaches—denoted by the green and blue intersecting arrows—will improve underpinning biochemical theory and identify candidate enzymes and pathways for the co-design of new physiological assays, as depicted in the bottom cartoon summarizing putative phosphorus uptake, metabolism and storage in phytoplankton (redrawn and simplified from Fig. 4 in Lin *et al*., 2016). The present cartoon is for a cyanobacterium, which in addition to the plasma membrane, also contains a semipermeable outer membrane (shown as a dotted line). Abbreviations: ADP = adenosine diphosphate; ATP = adenosine triphosphate; AP = alkaline phosphatase; Pi = inorganic phosphorus; PolyP = polyphosphate; gene names as described in Lin *et al*., 2016).

## LINKING PHYSIOLOGY AND OMICS: THE NEED FOR CO-DESIGN

We propose that physiological rates can bridge biogeochemistry and omics. Physiological rates quantify the integrated activity of proteins that drive marine biogeochemical cycles in units that modelers can use (Fig. 3). Research into the ocean’s N cycle reveals the potential of using the joint expertise of the physiology and omics communities (i.e. co-design) to guide future research (Fig. 1). We can extend this complementary approach to use omics datasets to develop new targeted physiological metrics that improve the parameterization of biogeochemical processes. Here, we explore the feasibility of co-design using case studies of the ocean P and Fe cycles that illustrate how physiological metrics may act as a “currency converter” to link omics datasets and biogeochemical models.

In the case of P, a lab study used proteomics and physiological metrics to explore the cumulative effect of five climate-change stressors on a subpolar diatom (Boyd *et al*., 2015). A central finding was that the effect of decreased nutrient supply in a future ocean was offset by warming. Proteomics revealed that a decreased need for P was driven by lower expression of P-containing proteins associated with translation (Fig. 3). Physiological metrics corroborated this finding, with lower cellular P quotas under warming. Hence, P quotas acted as a currency converter between protein synthesis and the biogeochemical cycle of P. They showed serendipitously a link between protein synthesis and P quotas. In the future, we must actively seek conceptual linkages, rather than uncovering them by chance. Better links from omics via physiology to biogeochemistry would benefit from input from the modeling and biogeochemical research communities.

Physiology was established earlier than omics or biogeochemistry, which begs the question: are we currently measuring the best physiological metrics to mesh omics with biogeochemistry? Two examples that begin to straddle the gaps between omics and physiology come from Saito *et al*. (2011) and Wu *et al*. (2019). The former revealed diel changes in the proteome, including Fe-metalloproteins involved in N_2_ fixation and photosynthesis of *Crocosphaera watsonii* resulting in more efficient use of Fe, which is essential for N_2_ fixation. In the latter case, protein expression and physiological metrics were coupled to examine the influence of Fe and manganese on *Phaeocystis antarctica*.

Although our current choice of physiological metrics needs urgent scrutiny, there is compelling evidence of the utility of long-established assays, such as those used to determine the macromolecular P content of cells from Liefer *et al*. (2019), for more innovative phytoplankton cellular P models (Inomura *et al*., 2020). But can we be inventive and use omics to interpret P physiology in a more holistic manner (Fig. 4)? Physiology can provide valuable insights, even when considering only a few components of the cellular P cycle. Imagine the progress if we developed better metrics jointly with omics (Feng *et al*., 2014; Lin *et al*., 2016). Therefore, the way ahead may be to use molecular biology to “reverse engineer” the most pertinent physiological metrics (Fig. 4). For example, a useful point of departure would be to select processes in which protein abundance correlates with quantifiable metabolic activity. It is likely that a range of approaches—from metabolic theory, ocean observations of key metabolites along with improved maps of cellular functioning from omics (e.g. iPath)—can help jointly refine the targeting of key biochemicals most suitable for reverse-engineering of better metrics. In some cases, for targeted research questions, the range of approaches presented as illustrative examples in Fig. 4 may need to be expanded upon or modified. Such co-design, in our opinion, will further facilitate the transition from lab- to field-based omics and will lead in the coming decades to incorporation of omics into biogeochemical models.

**Fig. 5:**
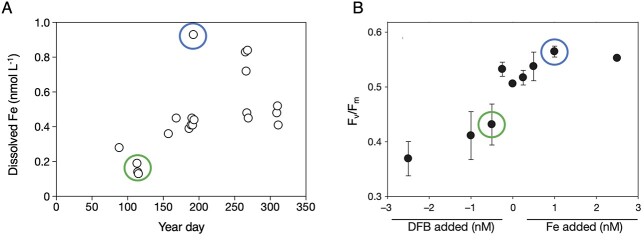
Utility of environmental context to define the present physiological status of cells in relation to prior oceanic conditions. (**A**) Dissolved Fe time series for the upper ocean in the subtropical Atlantic (BATS site) that reveals conspicuous aerosol Fe inputs (>0.5 nmol L^−1^) along with the influence of eddy activity (<0.3 nmol L^−1^) on dissolved Fe concentrations (Sedwick *et al*., 2020). (**B**) Photosynthetic efficiency of PSII (*F*_v_/*F*_m_) measured in deckboard incubation experiments “titrated” with dissolved Fe concentrations by either lowering bioavailable Fe using the fungal siderophore desferrioxamine B (DFB) or increasing it with chelated inorganic Fe addition (from Wilhelm *et al*., 2013). The circles denote putative linkages between chemical stocks and biological responses (blue = high Fe; green = low Fe).

The transition to field studies will face additional challenges that centre on how marine biota integrate environmental history (i.e. cellular status imposed by conditions encountered prior to sampling; Fig. 2) (Prairie *et al*., 2012; Deutschmann *et al*., 2021). This requires a multi-stranded approach. Placing the sampling locale in a wider environmental context will be necessary (Fig. 5A). For example, profiling robotic floats with multiple sensors are providing synoptic snapshots of spatial variability in ocean properties along with the prior seasonal dynamics of key resources such as nutrients (Claustre *et al*., 2021). It will also be essential to determine how such prior oceanic conditions set cellular status, for example the degree of Fe stress (Fig. 5B). An open question is whether the relationship between environmental forcing and cellular status is instantaneous or lagged (Fig. 2). Will such co-designed metrics reconcile a biological product with a chemical residual since different physiological metrics display a range of response times (Boyd *et al*., 2005; Baker *et al*., 2018), as do different omics layers (Waldbauer *et al*., 2012)? One promising approach to probe environmental history and cellular status is physiological titration, for example by manipulating Fe availability to contextualize cellular Fe status (Fig. 5B).

## CONCLUSIONS AND FUTURE DIRECTIONS

We conclude with recent field-leading examples from ocean sciences that seek to derive metabolic rates from omics, explored through the lens of biomedical sciences. Saito *et al*. (2020) conducted metaproteomic analysis on subsurface biota in the Tropical North Pacific to pinpoint commonly occurring enzymes. They reported that nitrite oxidoreductase associated with the bacterium *Nitrospina* was abundant in this stratum and explored whether they could estimate rates of nitrite oxidation using wide-ranging methods, including biochemistry (specific activity), physiology (Michaelis–Menten kinetics) and omics. Despite employing this innovative suite of approaches, derived rates ranged >200-fold, pointing to the need to develop targeted physiological assays (c.f. Fig. 4). There are also promising initial developments from the emergence of phenomenological models based on simple geochemical/taxonomic principles that yield phytoplankton growth rates assuming steady-state growth (McCain *et al*., 2021).

The latest developments in biomedical and model-system omics suggest that obtaining rates from omics is still under development. First, holistic investigations of well-characterized model organisms have tracked every metabolite and protein to generate enzyme-directed functional rates in the bacterium *Escherichia coli* (Taniguchi *et al*., 2010) and the yeast *Saccharomyces cerevisiae* (Ho *et al*., 2018), but this approach is restricted to the organisms for which the function of every gene and protein is known. Second, expression-fitness landscapes (linking enzyme expression with growth rate) have revealed that enzyme expression can have a “ripple” effect across layers of biological organization ranging from mechanistic, regulatory to systemic (Lalanne *et al*., 2021), which adds further complexity to deriving growth rates from enzymatic fluxes. Third, sophisticated microbiome studies (from cheese to the human gut) (Poceviciute and Ismagilov, 2019), which are more akin to oceanic microbial systems, reveal that there are still a high number of metabolic functions that remain uncharacterized (Price *et al*., 2018). Fourth, progress in tackling cell regulatory mechanisms using multiomic modeling has been made but requires complex computing using deep neural networks such as GEMS (Genome-scale metabolic models) (Okada and Kuroda, 2019).

These four categories of advanced well-resourced research point to challenges yet to be surmounted in obtaining physiological rates from omics for biomedical sciences. But, they also provide cautionary lessons for ocean sciences. In our opinion, it may be more straight-forward to co-design targeted physiological metrics that better link omics with marine biogeochemistry. We advocate for better communication across these research communities that could be readily facilitated through co-design workshops and other forums to ascertain the best ways to reverse-engineer a new generation of physiological metrics, in tandem with the development of high-throughput technologies to promote “co-measurement” (Fig. 4), that better exploit the power of molecular biology to answer the most pressing questions in ocean sciences.

## Dedication

We dedicate this article to the late Paul J. Harrison, a phytoplankton physiologist who influenced and inspired generations of scientists, including several of the authors.
